# The evaluation of renal ischaemic damage: the value of CD10 monoclonal antibody staining and of biochemical assessments of tissue viability

**DOI:** 10.1186/1472-6890-7-5

**Published:** 2007-05-26

**Authors:** S Tagboto, A Paul Griffiths

**Affiliations:** 1 Department of Nephrology, University Hospital of North Staffordshire, Royal Infirmary, Princes Road, Hartshill, Stoke on Trent ST4 7NL, UK; 2 Department of Histopathology, Morriston Hospital, Morriston, Swansea SA6 6NL, UK

## Abstract

**Background:**

It is well recognised that there is often a disparity between the structural changes observed in the kidney following renal injury and the function of the organ. For this reason, we carried out studies to explore possible means of studying and quantifying the severity of renal ischaemic damage using a laboratory model.

**Methods:**

To do this, freshly isolated rabbit kidney tissue was subjected to warm (37°C) or cold (1°C) ischaemia for 20 hours. Following this, the tissue was stained using Haematoxylin and Eosin (H+E), Periodic Schiff reagent (PAS) and the novel monoclonal antibody CD10 stain. Additionally, ischaemic damage to the kidneys was assessed by biochemical tests of tissue viability using formazan-based colorimetry.

**Results:**

CD 10 antibody intensely stained the brush border of control kidney tissue with mild or no cytoplasmic staining. Cell injury was accompanied by a redistribution of CD10 into the lumen and cell cytoplasm. There was good correlation between a score of histological damage using the CD 10 monoclonal antibody stain and the biochemical assessment of viability. Similarly, a score of histological damage using traditional PAS staining correlated well with that using the CD10 antibody stain.

In particular, the biochemical assay and the monoclonal antibody staining techniques were able to demonstrate the efficacy of Soltran (this solution is used cold to preserve freshly isolated human kidneys prior to transplantation) in preserving renal tissue at cold temperatures compared to other randomly selected solutions.

**Conclusion:**

We conclude that the techniques described using the CD10 monoclonal antibody stain may be helpful in the diagnosis and assessment of ischaemic renal damage. In addition, biochemical tests of viability may have an important role in routine histopathological work by giving additional information about cellular viability which may have implications on the function of the organ.

## Background

Renal histopathology is an invaluable clinical tool. It is used to diagnose the cause of dysfunction of native and transplanted kidneys and to offer predictions about the likely course of renal disease. Nevertheless, studies on acute tubular necrosis and other renal diseases have consistently reported that there is often disparity between structural changes in the kidney and function of the organ [1]. This study was carried out to explore novel means of studying and quantifying the severity of renal ischaemic damage using a laboratory model.

We induced ischaemic damage by maintaining freshly obtained thin cylindrical kidney biopsy specimens under tissue culture conditions. Kidney biopsy cores were thus subjected to warm (37°C) or cold (1°C) ischaemia for 20 hours. The use of randomly selected solutions and two incubation temperatures permitted the development of different degrees of ischaemic damage. Following this, biopsy cores were stained with Haematoxylin and Eosin (H+E), Periodic Schiff reagent (PAS) or the monoclonal antibody CD10. The histological appearances were then compared.

Ischaemic damage to the kidneys was also assessed by biochemical techniques using formazan-based colorimetry. This assay has been shown to be useful in the assessment and quantification of the viability of organisms, cells or tissues in experimental studies [2,3] but has been applied for the first time in this manner to renal tissue. This work differs from traditional studies that have generally used the whole organ *in vivo *or in isolation in studies that are more cumbersome to carry out [4].

We have described the distribution of the CD 10 antibody stain in kidney biopsy cores following experimentally induced ischaemia. We have additionally compared a score of ischaemic damage using this stain with a score using PAS and using a biochemical assessment of tissue viability.

## Methods

### Kidneys

The kidneys used in this study were obtained from laboratory rabbits which were the control animals in other experimental research. These kidneys were donated to this study. The kidneys were removed immediately after death (within 5 minutes). Following this, renal biopsy cores were removed using a 16 gauge (1.6 mm thick) biopsy gun (Single Action Biopsy Device, K7/SABD-1615-T, Kimal plc, Arundel Road, Uxbridge, Middlesex, England, UB8 2SA). The biopsy cores were then cut to a standard length of 5 mm.

Biopsy cores thus obtained (total 12–15 minutes of warm ischemia) were immediately placed randomly and individually in the relevant pre-cooled (1°C) or pre-warmed (37°C) solution and maintained in a 24 well flat-bottomed plate for 20 hours.

### Culture system

5 mm long kidney biopsy samples were placed singly in each well of a 24 well flat-bottomed tissue culture plate (Becton Dickinson, UK).

In an attempt to induce varying degrees of ischaemic damage, we incubated the biopsy cores in 1.8 ml of various randomly selected solutions. These included the commercially available kidney preservative solution, Soltran (Baxter), selected laboratory media including Eagles Minimum Essential Medium (MEM), newborn calf serum (NCS), or both {with or without a feeder layer of monkey kidney cells (LLCMK2) (Gibco)}, deionised water or phosphate buffered saline (PBS).

Samples were maintained in an atmosphere of 5% CO_2 _in air in a Heraeus EK/O2 tissue culture incubator at 37°C or in air at 1°C for 20 hours.

At least 6 replicate biopsy samples were maintained under each of the culture conditions. Four were subsequently used for biochemical assays and the rest for histology.

### MTT reduction assay

This technique is based on the formation of a blue water-soluble formazan compound from the pale yellow tetrazolium salt 3-(4, 5 dimethylthiazol-2-yl)-2, 5-diphenyltetrazolium bromide (MTT) after reduction by dehydrogenase enzymes, principally NADH and NADPH, in the mitochondria of living tissue. The intensity of colour formed gives a measure of tissue viability.

Following the 20 hour incubation, kidney biopsy samples were incubated singly in 1 ml of pre-warmed (37°C) MTT solution (0.5 mg/ml) in wells of a 24 well flat-bottomed plate. After 30 minutes, the kidney samples were removed and placed individually in wells of a 48 well flat-bottomed plate containing 400 μl of dimethylsulphoxide (DMSO) and left at room temperature for 1 hour, thus solubilising the MTT formed within the biopsy samples. The plate was occasionally agitated to disperse the colour evenly. The absorbance of 200 μl of the resulting formazan solution was determined at 490 nm in a multi-well scanning spectrophotometer (MR 700, Dynatech laboratories) relative to a DMSO blank.

### Histology and immunocytochemistry

Following the 20 hour incubation, cores of renal tissue were fixed in 10% formalin prior to processing and embedding in paraffin wax. 4 μm sections were stained with Haematoxylin and Eosin (H+E), Periodic Schiff reagent (PAS) and the monoclonal antibody CD10. PAS and CD10 were used to demonstrate the integrity of the tubular brush border.

Slides stained with PAS were analysed according to four histological criteria.

1. Preservation of PAS-stained brush border.

2. Preservation of cell boundaries (ie. cell integrity).

3. Nuclear detail with top score where the majority have a clear nuclear membrane and nucleolus.

4. Adhesion of cells to the basement membrane.

**Figure 1:**
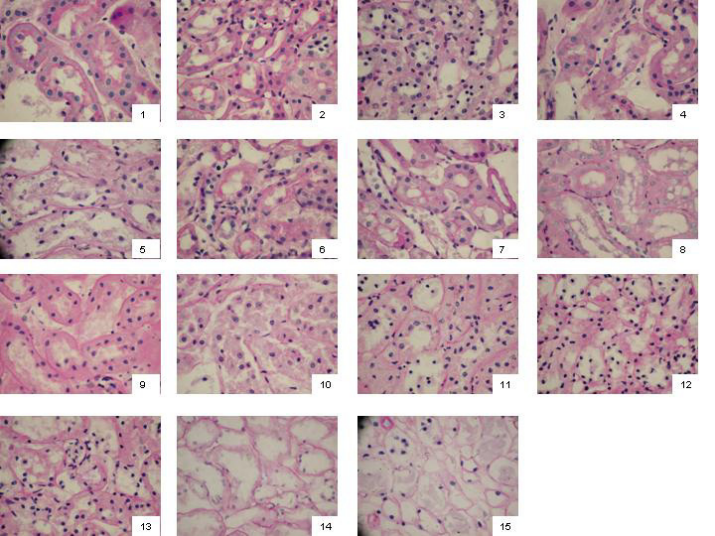
**Kidney biopsy specimens stained with PAS**. See text and table for histology scores and for description of solution and temperature used to preserve kidneys.

Each slide was scored according to the above criteria as 0 (none), 1 (poor), 2 (moderate) or 3 (excellent). The scores for each criterion were than totalled. This resulted in a final histological score of between 12 (excellent renal preservation) and 0 (poor renal preservation) (see Fig 1 & Table 1).

Additionally, the CD10 monoclonal antibody (Vision Biosystems, previously Novocastra) was used at a dilution of 1:800, with mild antigen retrieval (30 mins in CCI buffer/citric acid), incubated with antibody for 30 mins at 40°C and visualised with an I-View Detection Kit which includes a biotin blocker. Slides were processed using an automated staining machine (Benchmark, Ventana). This provided an intense stain with strong contrast.

**Figure 2:**
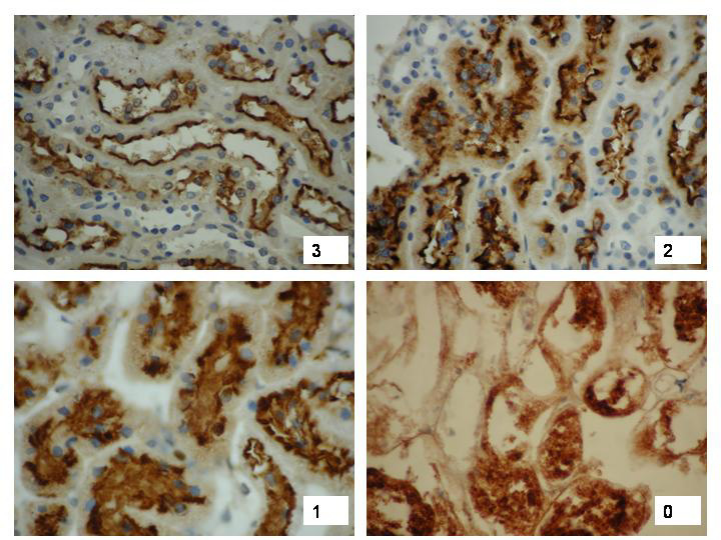
**Patterns of staining of Kidney Biopsy specimens stained with CD 10 antibody**. 3. Well defined, intensely stained brush border with mild or no cytoplasmic staining (immunostaining score = 3). 2. Brush border relatively well-defined, but some loss into the lumen and more cytoplasmic staining than 1. (immunostaining score = 2). 1. Loss of distinct brush border staining, staining of luminal contents and heavy cytoplasmic staining. (immunostaining score = 1). 0. Disrupted cells with diffuse staining and no definite cytoplasmic border. (immunostaining score = 0)

Cortical areas were graded 3 (excellent renal preservation) to 0 (poor renal preservation) according to the predominant pattern of CD10 staining (Fig 2).

The 2 scoring systems were compared (Table 1).

## Results

Kidney biopsy cores were incubated as previously described. At the end of the incubation period, the viability of the biopsy samples was assessed by MTT assay (Table 1). Control cores were not incubated in preservative solution before the assay was carried out.

The amount of formazan formed in the cores (4 replicates) following incubation for 20 hours (numbered 1–7 & 9–15, Table 1) were compared to that formed in control samples (numbered 8, Table 1). Mean optical density readings were taken and expressed as % reduction compared to control readings.

The biopsy cores were maintained at either 1°C (1–7) or 37°C (9–15), under seven different culture conditions. Formazan formation was inhibited to a greater degree following incubation at the higher temperature (Fig 2). This indicates that the lower temperature (1°C) protects tissue viability.

**Figure 3:**
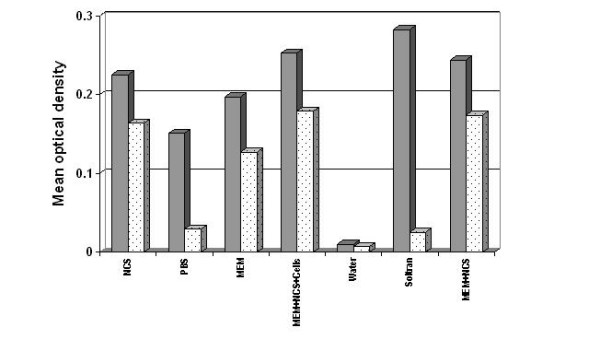
The viability of kidney biopsy tissue assessed by MTT assay. Mean optical density readings following incubation in preservative solutions at 1°C (shaded boxes) or 37°C (dotted boxes). See text for abbreviations.

At 1°C, the commercially available preparation Soltran offered the best biochemical preservation of tissue viability (Fig 3), in keeping with research evidence concerning the efficacy of this product in kidney preservation prior to renal transplantation.

The incubation of biopsy samples in water offered the least renal preservation at all temperatures. This is in keeping with studies in the literature that have demonstrated that renal preservation is not satisfactorily achieved at low osmolarity. The osmolarity of the commercially available fluids, University of Wisconsin solution and Soltran used in renal transplantation are 320 and 486 mOsm respectively. The osmolarity of serum by comparison is 285–295 mOsm.

Incubation in tissue culture medium (MEM) or in newborn calf serum (NCS) or both offered superior tissue preservation to that of the simple electrolyte solution PBS at either temperature.

The addition of a feeder cell layer to MEM+NCS did not offer significant additional renal preservation.

The morphological sequelae of ischaemia in the isolated renal biopsy cores were similar to those previously described *in vivo*. Kidneys subjected to cold ischaemia showed only isolated areas of mild brush border damage except when the preservative used was deionised water. Kidneys subjected to warm ischaemia showed marked morphological damage with dilatation of tubules and widespread loss of the brush border.

**Figure 4:**
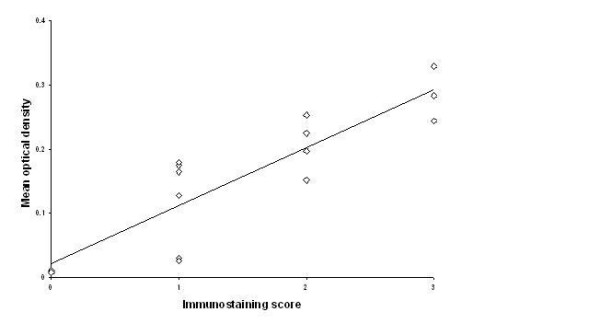
The viabililty of renal biopsy samples assessed by histopathological damage using immunostaining with CD10 antibody and by MTT reduction following incubation in preservative solution for 20 hours.

Higher immunostaining scores correlated positively with higher histology scores (Correlation coefficient 0.800, P < 0.001) and with better biochemical viability assessed by MTT reduction (Correlation coefficient 0.872, P < 0.001) (Fig. 4). Higher histology scores also correlated with better biochemical viability (Correlation coefficient 0.749, P < 0.001)

## Discussion

This study has explored the possibility of employing a new stain (CD 10 antibody) and a test of tissue viability (formazan based colorimetry) to study and quantifying renal ischaemia.

CD 10 (also called neutral peptidase, neprilysin or CALLA), is a 100 KD cell surface metalloproteinase. It inactivates a variety of biologically active peptides thus limiting the response of cells following stimulation by peptide hormones. It was originally identified as a common acute lymphoblastic leukaemia antigen (CALLA) and initially thought to be tumour specific. However, it is now known to be present in other haematological malignancies. The utility of the CD 10 monoclonal antibody in the diagnosis of these malignancies is now well established [5]. Recent studies have shown that CD10 is expressed on the surface of a wide variety of normal and neoplastic cells. In particular, there is high expression on the brush border of normal renal tubules and glomerular capillary walls (McIntosh *et al*., 1999) and on the cytoplasmic cell membranes of human renal cell carcinoma [6,7].

Renal ischaemia disrupts the cytoskeletal architecture resulting in relocation of apical and basolateral membrane-specific proteins such as ankyrin, fodrin and Na^+ ^K^+ ^-ATPase. In particular, the loss of polarity and blebbing of the brush border can be demonstrated after as little as 5 minutes ATP depletion [1]. Since the CD 10 monoclonal antibody localises to the brush border, we felt that it might be useful to study its distribution in renal ischaemia. This monoclonal antibody had already been optimised for routine diagnostic use in our histopathology department for lymphoma typing.

The MTT reduction assay relies on the reduction of this compound by dehydrogenase enzymes, principally NADP and NADPH, in the mitochondria of viable cells and tissue. This indirectly measures the integrity of glucose metabolism through glycolysis and the Krebs cycle and has been shown to be a useful measure of tissue viability [3].

In this study, cell injury was accompanied by a redistribution of CD10 antibody into the lumen and the cell cytoplasm, and a reduction in formazan formation from the biopsy cores. There was a significant correlation between scores of ischemic damage following routine histology, biochemical scores of viability and CD 10 antibody staining. The use of biochemical tests and CD 10 antibody staining offers the possibility of complementing routine histology in the diagnosis of acute ischaemic renal damage.

Formazan based colorimetry has been used by one of the authors for several years to assess damage to parasitic helminths following trials of chemotherapeutic agents [8]. Furthermore, prior to these studies, we had demonstrated that formazan production from isolated kidney tissue is reduced in a time and temperature dependent fashion [9]; in keeping with predictions from other experimental work [4]. We therefore decided to use this assay to confirm the proof of principle in this pilot study. Nevertheless, it remains important to carry out further studies comparing formazan colorimetry with kidney function assessed by glomerular filtration rate. Additionally, there are many other biochemical assays (radioactive glucose uptake, leucine uptake, CO2 evolution, adenine uptake and leakage and lactate output) which have been employed in other scientific disciplines to assess the viability of cells or microbes [2,3]. In future studies, it will be essential to compare predictions offered by these different techniques to whole organ function.

Alternate methods of assessing kidney viability prior to transplantation are being researched including the use of flow characteristics combined with enzyme leakage from machine perfused kidneys [10,11]. The tests we have described may also be helpful in predicting the suitability of borderline kidneys particularly from non-heart beating donors prior to renal transplantation, compared to current methods mainly involving visual inspection.

CD10 antibody offers the possibility of complementing routine histology in the diagnosis of acute ischaemic renal damage, although we would see it as being probably more relevant in research applications. CD10 stains the tubular brush border more intensely than PAS and might lend itself to quantification by image analysis, either in immunoperoxidase or fluorescent preparations. The value of redistribution of other relevant proteins such as ankyrin and fodrin might be explored in the future.

## Conclusion

The use of biochemical tests and CD 10 antibody staining offers the possibility of complementing routine histology in the diagnosis of acute ischaemic renal damage. These studies open up new possibilities for studying the effect of ischaemia on the kidneys.

## Competing interests

The author(s) declare that they have no competing interests.

## Authors' contributions

ST conceived this research and carried out the biochemical tests of viability. APG arranged the PAS and CD 10 antibody stain and carried out the histological scores of viability. ST and APG were involved in drafting and revising the manuscript. All authors read and approved the final manuscript.

## Pre-publication history

The pre-publication history for this paper can be accessed here:



## Figures and Tables

**Table 1:** The effect of exposure to preservative solution for 20 hours on the viability of renal biopsy tissue assessed by MTT reduction and a histology score following CD 10 antibody staining. See text for abbreviations.

Preservative solution	Temp. of incubation	Duration of incubation	Mean optical density readings	% reduction in formazan formation	Histology score (PAS)	Immuno-staining score (CD 10)
1. NCS	1°C	20 hours	0.225	31.60%	12	2
2. PBS	1°C	20 hours	0.151	54.10%	12	2
3. MEM	1°C	20 hours	0.197	40.10%	7	2
4. MEM+NCS+Cells	1°C	20 hours	0.253	23.10%	10	2
5. Water	1°C	20 hours	0.01	97.00%	4	0
6. Soltran	1°C	20 hours	0.283	14.00%	8	3
7. MEM+NCS	1°C	20 hours	0.244	25.80%	9	3
**8. None (Control)**	**37°C**	**0 minutes**	**0.329**	**-**	**11**	**3**
9. NCS	37°C	20 hours	0.164	50.20%	5	1
10. PBS	37°C	20 hours	0.029	91.20%	4	1
11. MEM	37°C	20 hours	0.127	61.40%	6	1
12. MEM+NCS	37°C	20 hours	0.174	47.10%	4	1
13. MEM+NCS+Cells	37°C	20 hours	0.179	45.60%	4	1
14. Water	37°C	20 hours	0.007	97.90%	0	0
15. Soltran	37°C	20 hours	0.025	92.40%	3	1
